# A Robust Speaker Identification System Using the Responses from a Model of the Auditory Periphery

**DOI:** 10.1371/journal.pone.0158520

**Published:** 2016-07-08

**Authors:** Md. Atiqul Islam, Wissam A. Jassim, Ng Siew Cheok, Muhammad Shamsul Arefeen Zilany

**Affiliations:** Department of Biomedical Engineering, Faculty of Engineering, University of Malaya, Kuala Lumpur, 50603, Malaysia; University of California, Irvine, UNITED STATES

## Abstract

Speaker identification under noisy conditions is one of the challenging topics in the field of speech processing applications. Motivated by the fact that the neural responses are robust against noise, this paper proposes a new speaker identification system using 2-D neurograms constructed from the responses of a physiologically-based computational model of the auditory periphery. The responses of auditory-nerve fibers for a wide range of characteristic frequency were simulated to speech signals to construct neurograms. The neurogram coefficients were trained using the well-known Gaussian mixture model-universal background model classification technique to generate an identity model for each speaker. In this study, three text-independent and one text-dependent speaker databases were employed to test the identification performance of the proposed method. Also, the robustness of the proposed method was investigated using speech signals distorted by three types of noise such as the white Gaussian, pink, and street noises with different signal-to-noise ratios. The identification results of the proposed neural-response-based method were compared to the performances of the traditional speaker identification methods using features such as the Mel-frequency cepstral coefficients, Gamma-tone frequency cepstral coefficients and frequency domain linear prediction. Although the classification accuracy achieved by the proposed method was comparable to the performance of those traditional techniques in quiet, the new feature was found to provide lower error rates of classification under noisy environments.

## Introduction

Speaker recognition is a biometric modality that uses underlying speech information to determine the identity of the speaker. Speaker recognition is employed for a wide range of applications such as in banking over a telephone network, voice dialing, voice mail, database access services, telephone shopping, security control for confidential information, remote access to computers, forensic tests, and information and reservation services. The application of speaker recognition can be divided into two parts: speaker identification and speaker verification. Speaker identification (SI) is to determine the identity of an unknown speaker based on his/her speech utterances whereas speaker verification is to use the voice to verify a certain identity claimed by the speaker [1]. Recognition of speakers is done based on text-dependent and text-independent speech samples. The text-dependent system requires the speakers saying exactly the same utterance (word, phrase, or sentence) whereas the identification without constraints on the speech content represents the text-independent identification system. A substantial research has been done on speaker recognition under diverse background conditions over the last few decades: from vector quantization (VQ)-based SI system [2] to adapted Gaussian Mixture Model (GMM)-based system [3], and very recently speech factor analysis-based i-vector frameworks [4]. In general, the features from the speech signal for speaker recognition are extracted by modeling the human voice production system (such as linear prediction cepstral coefficient, LPCC [5]) or from the responses of human auditory system. Human listeners are capable of recognizing speakers in noisy environments, while most of the traditional speaker recognition systems do not perform well in the presence of noise [6]. Unlike traditional methods in which features are extracted from the properties of the acoustic signal, this study proposes a speaker identification technique using neural responses from a physiologically-based computational model of the auditory periphery.

For speaker recognition, it is important to extract features from each frame which can capture the speaker-specific characteristics. The short-time features such as the Mel-frequency cepstral coefficients (MFCC) and perceptual linear predictive (PLP) coefficients are widely used for speaker recognition algorithms. The traditional MFCC-based system achieved almost 100% classification accuracy in clean condition [7, 8]. However, the performance of these acoustic-property-based methods degrades substantially for speech signals under channel variations induced by the handset or microphones as well as for environmental or background distortions [6]. In recent years, efforts have been made aiming to extract features by removing the noise from the speaker characteristic information directly such as cepstral mean normalization [9], RASTA processing [10], warping methods [11], and robust parameterizations [12]. However, these methods have limited effectiveness against non-linear channel effects and non-stationary additive distortions. Recently, the missing data approach has been employed to design a robust speaker identification system [13] which has a conceptual relationship with the human auditory system and its ability to process corrupted speech signals [14]. Conceptually, missing data processing is based on the idea that the noise-induced degradation can be reduced by identifying the speech and noise dominant parts of the corrupted speech in the time-frequency (T-F) representation, and thus the recognition perfromance could be substantially improved.

In 2010, Li and Huang proposed a new front-end speech feature, cochlear filter cepstral coefficient (CFCC) [15], that has been extracted by emulating the human peripheral hearing system and is shown to achieve an improved perfromance in noise robustness under mismatched training and testing conditions. However, the auditory filters used in this study are linear, and some of the parameters of this method are database-dependent. Recently, another feature, frequency domain linear prediction (FDLP) coefficient [16], has been proposed using 2-D autoregressive (AR) models on the high energy peaks of the speech signal in the T-F domain. It was reported that FDLP feature provided ~30% improvements in speaker recognition performance under noisy conditions compared to using the baseline MFCC feature. Also, Zhao et al. [17] has reported a substantial improvement in speaker identification performance using Gammatone frequency cepstral coefficients (GFCCs) when a computational auditory scene analysis technique is employed to produce a binary T-F mask, especially under noisy conditions.

In the present study, the proposed feature was derived directly from the responses of a computational model of the auditory periphery to speech signals. The proposed method differs from all previous auditory-model-based methods in that a more complete and physiologically-based model of the auditory periphery is employed in this study [18]. The auditory-nerve (AN) model developed by Zilany and colleagues has been extensively validated against a wide range of physiological recordings from the mammalian peripheral auditory system. The model can successfully replicate most of the nonlinear phenomena observed at different level of the auditory periphery (e.g., in the cochlea, inner-hair-cell (IHC), IHC-AN synapse, and AN fibers). These phenomena include the nonlinear tuning, compression, two-tone suppression, level-dependent rate and phase responses, shift in the best frequency with level, adaptation, and several high-level effects [18, 19, 20]. The model responses have been tested to both simple (e.g., tone) and complex stimuli (e.g., speech) for a wide range of frequency and intensity spanning the dynamic range of hearing. Thus the model is ready to be used for exploring the neural mechanisms (i.e., feature representation) for robust identification in different tasks including speaker recognition under diverse background conditions.

It is well-known that the features derived directly from the acoustic signals are very sensitive to noise, and thus the performance of the speaker recognition systems under noisy conditions declines sharply. On the other hand, neural responses are robust against noise due to the phase-locking property of the neuron [21], i.e., the neurons fire preferentially at a certain phase of the input stimulus (up to ~ 4 kHz at the level of the AN), even when noise is added to the acoustic signal. Human behavioral responses are also robust under diverse background noise conditions. Phase-locking also enables to extract inter-aural time differences down to a few microseconds for sound localization and may also be crucial in pitch perception, which is the salient feature of many speech vocalizations. It has also been reported that as the sound level increases, the formants of a vowel sound dominate the responses of AN fibers over a larger characteristic frequency (CF) region, and the responses of fibers with CFs near a formant frequency are captured by the largest harmonic near the formant, meaning that they show phase locking only to that harmonic [21]. It is this capture of responses by the formants, termed as the synchrony capture, that makes temporal representations of spectral shape very robust in the neural responses [22]. In response to a vowel sound at higher presentation levels, AN responses show the loss of synchrony capture by the second formant whereas synchrony to the first formant increases [23]. The current study was motivated by the fact that the AN model used in this study captures all of these observed phenomena in the physiological responses of the AN fibers [19, 24]. These properties of the model are expected to improve the proposed system’s robustness to noise.

Several speech intelligibility metrics have been proposed based on the responses of this model such as neurogram similarity index [25] and neurogram orthogonal polynomial measure (NOPM) [26]. The intelligibility scores predicted by NSIM and NOPM showed a good regression with the subjective scores for listeners with normal hearing and also for people with hearing loss under diverse background noise conditions such as with white Gaussian noise, speech-shaped noise and other environmental noises. Motivated by these results, a text-dependent speaker identification and verification systems were previously developed based on the responses of the model of the AN [27, 28], and a substantial improvement in performance was achieved over conventional systems.

The input to the AN model is the acoustic signal in the time domain, and the output is the response of an AN fiber with a particular characteristic frequency (CF) in terms of spike train sequence (i.e., discharge timings). By simulating the responses of a range of CFs, the 2D time-frequency representation, referred to as neurogram, is constructed. In this study, the neurogram coefficients were used as feature to determine the identity of unknown speakers. It is expected that the neurogram feature encode sufficient and important information about the speaker that would be crucial for recognition. In order to benchmark against some recently reported features, the proposed neural-feature-based system’s performance was compared to the performances of the conventional MFCC-, GFCC- and FDLP-based systems. Since the focus of this study was to evaluate the effectiveness of the feature in speaker identification (especially under noisy conditions), no effort was made to add any additional processing step (such as de-noising techniques) to improve the systems’ performance.

## Methodology

**Fig 1:**
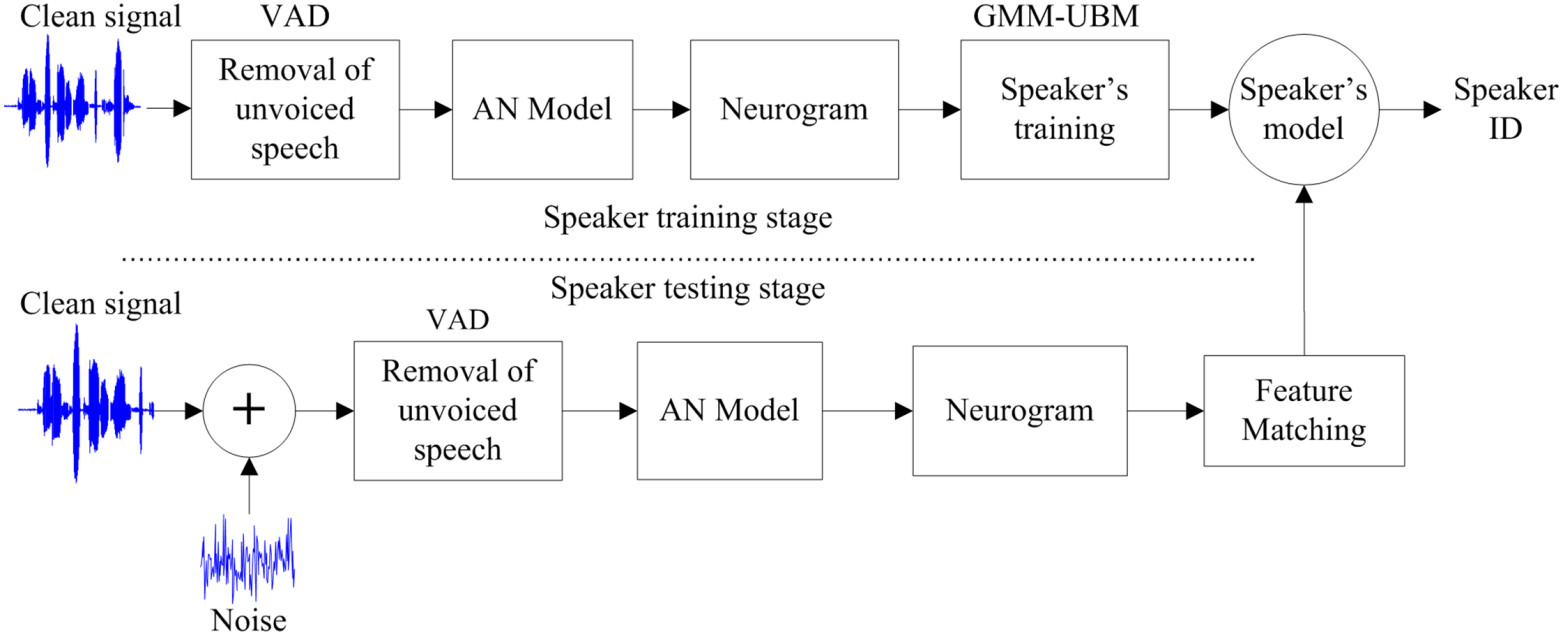
Block diagram of the proposed speaker identification system.

Fig 1 shows the block diagram of the proposed neural-response-based speaker identification system. In the training stage, the processed speech signal was applied to the model of the auditory nerve (AN) to generate the neurogram. The well-known Gaussian mixture model-universal background model (GMM-UBM) classifier was used to train the neurogram coefficients for each speaker, and the resulting model was saved for identification. In the testing stage, the extracted features of a test signal (speech of an unknown speaker) were used as an input to each of the speakers’ model. The model that provided maximum probability measure was identified as the speaker of the test sample. The performance of the proposed method was evaluated in clean and under noisy conditions. The following subsections briefly describe the computational procedure of each step of the proposed method.

### Pre-Processing

As a pre-processing step, the voice activity detector (VAD) algorithm [29] was used to remove the silence periods from speech signals. This algorithm also detected the unvoiced signal (especially with very low energies) indices of the input speech signal and removed those to provide voiced signal output.

### Model of the Auditory Nerve (AN)

The computational AN model developed by Zilany and colleagues [19] serves as a useful tool for understanding the underlying mechanical and physiological processes in the peripheral auditory system. The schematic block diagram of this AN model is provided in Fig 1 in [19]. Each block of the AN model represents a phenomenological description of the major functional components of the auditory periphery from the middle ear to the auditory nerve. The input to the model is an instantaneous pressure waveform in Pascal, and the output is the spike times. The first stage of the model filters the input signal to simulate the response properties of the middle ear. After the middle ear module, the model splits into three paths. A narrowband component 1 (C1) filter mimics the response properties of the basilar membrane (BM). The feed-forward control-path regulates the gain and bandwidth of the C1 filter to account for level-dependent properties associated with the outer hair cells (OHCs) such as compression, suppression, and nonlinear phase responses in the cochlea. The C1 and component 2 (C2) filters in the signal path interact to account for the effects associated with the AN responses at high sound levels such as the peak splitting and the C1/C2 transition [30]. The third stage simulates inner-hair-cell (IHC) mechanisms with a static nonlinearity followed by a fifth-order low-pass filter. The IHC output drives the model for IHC-AN synapse which includes exponential as well as power-law adaptations [20]. The model synapse output represents the probability of instantaneous discharge rate of AN fibers as a function of time which was used in this study to construct neurograms. Finally, the discharge times are produced by a renewal process that includes refractory effects.

In light of the current debate on human cochlear tuning [31, 32], some parameters of the most recent version of this model were adjusted to better match human anatomy and physiology. These modifications included changes to the middle-ear filter transfer function [33], the basilar membrane (BM) distance-frequency map [34] that determines the frequency-offset of the control path, and the BM frequency-tuning as a function of characteristic frequency [35, 36]. The sharpness of tuning was validated in a way that is consistent with the methodologies utilized in the literature to obtain the estimates from human subjects. Furthermore, changes in the model’s cochlear response delay because of the modified tuning were shown to be in agreement with estimates of cochlear delays in human subjects [37].

In this study, the neural responses were simulated to speech signals for 25 CFs logarithmically spaced between 250 and 4000 Hz. The responses of higher CFs (> 4 kHz) were not included in the proposed method, because the model synapse output becomes dominated by the dc value (constant) and the synchrony goes to a very lower value. The speech signal (original or noisy) was re-sampled to 100 kHz to apply as an input to the AN model. It is to be noted that the high sampling rate does not produce any frequency component beyond half of the sampling frequency of the original input signal, but it was required in order to faithfully replicate the frequency response properties of different parts of the AN model [20, 38]. The output at each AN fiber represents the instantaeous discharge rates in response to the input acoustic signal. For each CF, three types of spontaneous rates (SR) of fibers (high, medium and low) were considered in this study. Consistent with the distribution of SR of AN fibers [39], the maximum weight (0.6) was given to high SR fibers, and the weight given to medium and low SR fibers was 0.2 each. The neural responses for each CF were then binned with a 100 μs bin-width, and then a Hamming window of 42 ms was applied with a 60% overlap among adjacent frames to smooth the neural responses (i.e., the effective frame length was 25.2 ms). The mean value of each frame was used as a feature in the proposed method.

### Neurogram and the Feature Dimension

**Fig 2:**
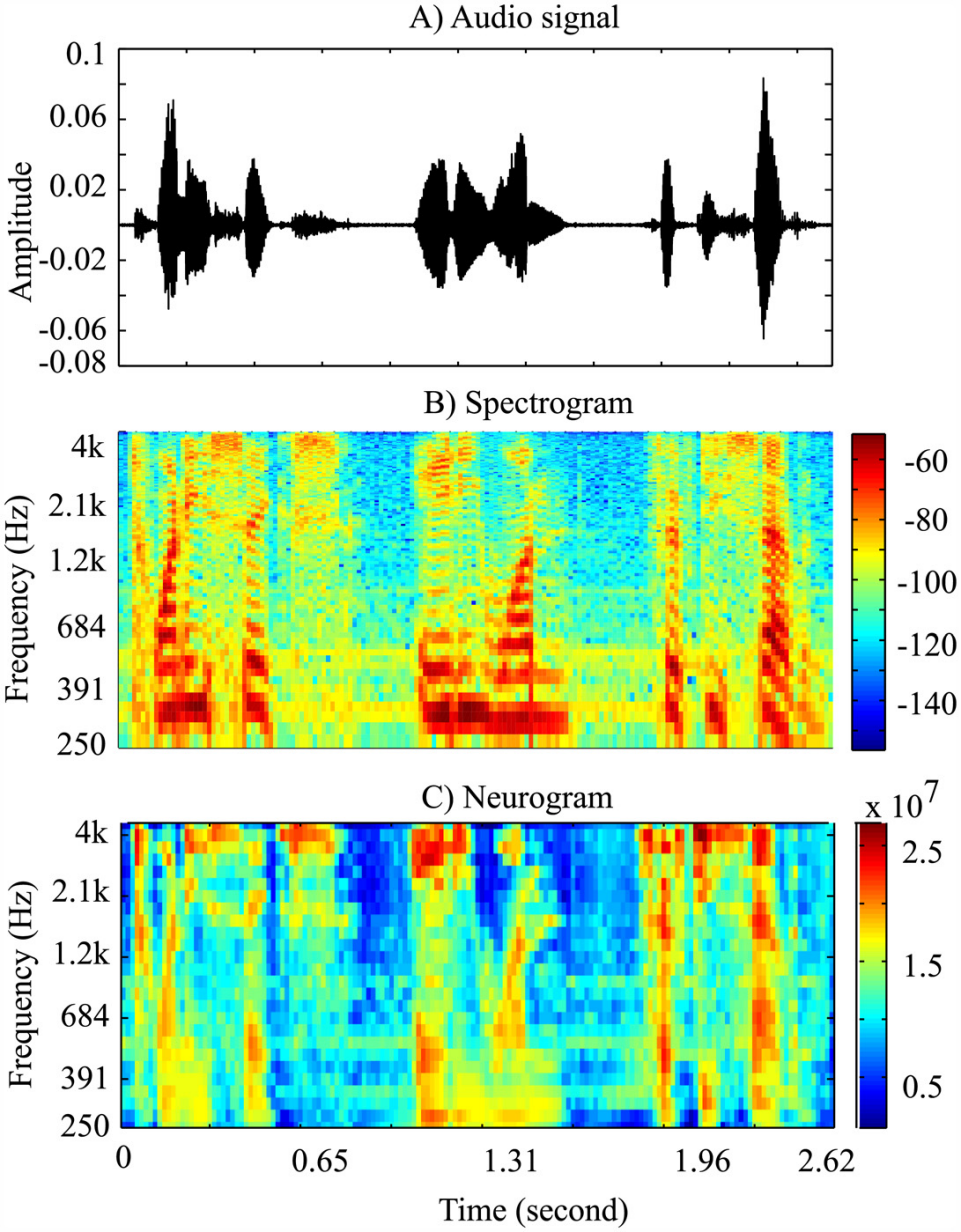
Time-frequency representation of the speech signal. (A) a typical speech waveform taken from the YOHO database (to produce spectrogram and neurogram of that signal), (B) the corresponding spectrogram responses, and (C) the respective neurogram responses.

Neurogram is a 2-D time-frequency representation which was constructed by combining the neural responses (i.e., feature) from 25 AN fibers. Fig 2 shows an example spectrogram and neurogram plots in response to a typical speech signal taken from the YOHO dataset. In this study, the neurogram coefficients were extracted for each speaker to be used as a feature for identification. The average size of the neurogram over three databases (considering all speech signals) was 190 × 25, where the number of frames was 190, and the number of AN fibers was 25.

**Fig 3:**
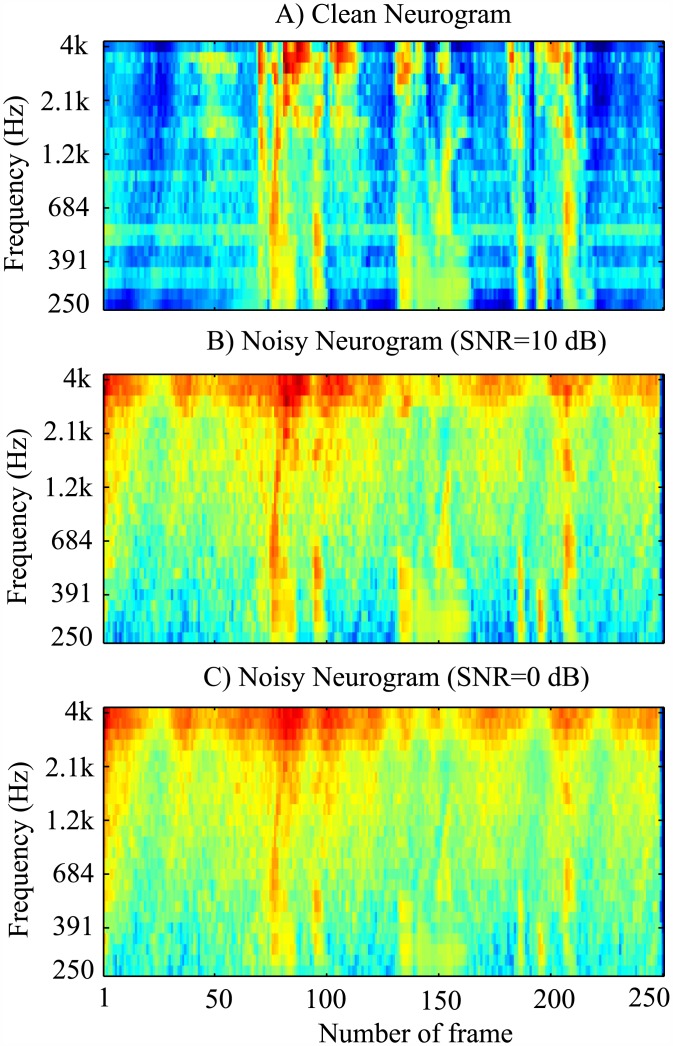
Illustration of the effects of noise on the neural responses. Neurogram responses are shown for a typical speech signal taken from the YOHO dataset. The neurogram to the clean speech signal is shown in the panel A, and the two neurograms in response to speech signal distorted by two levels of white Gaussian noise are shown in panels B (10 dB SNR) and C (0 dB SNR).

**Fig 4:**
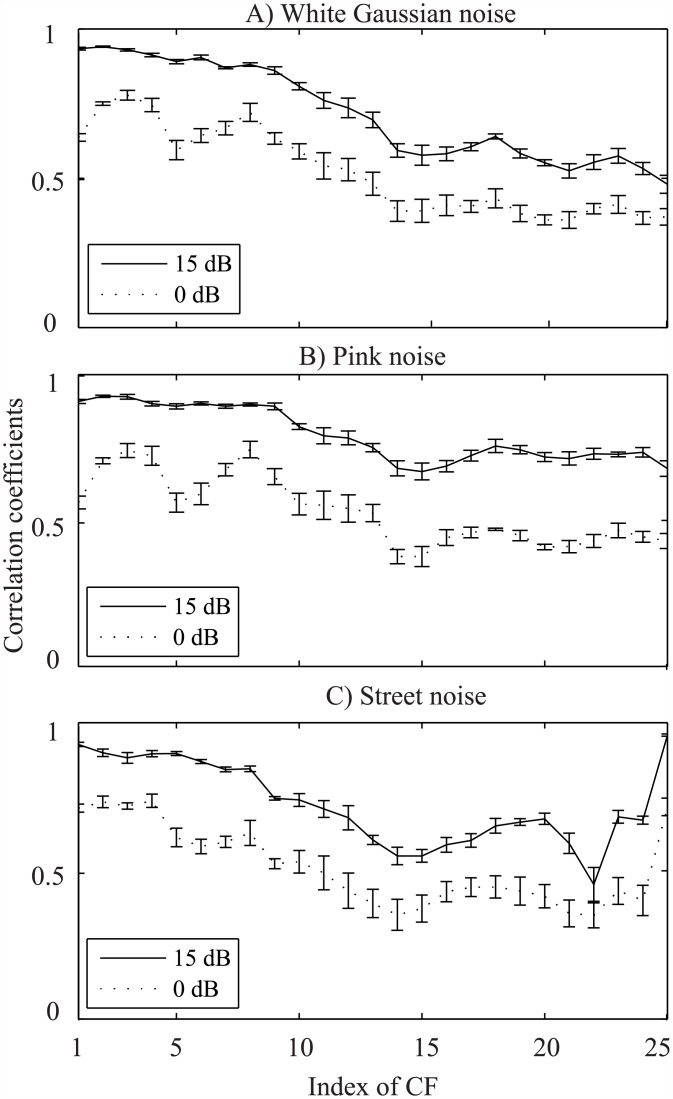
Illustration of the effects of noise on the neural responses. Neural responses were simulated to five clean speech signals taken from the YOHO database and to the corresponding noisy signals for SNRs of 0 and 15 dB with three types of noise: white Gaussian noise, pink and street noise. The correlation coefficients were calculated between the clean and the corresponding noisy signals for each CF, and the results are shown for 25 CFs (up to a phase-locking range of ~4 kHz). Panel A shows the mean and standard deviation of the correlation coefficients calculated for white Gaussian noise, and the corresponding results for pink and street noise are shown in panel B and C, respectively.

In order to illustrate the effects of noise on the neurogram responses, Fig 3A shows the neurogram plot for a clean speech signal taken from the YOHO dataset, and the neurograms for the same speech signal distorted by white Gaussian noise with SNRs of 10 and 0 dB are shown in Fig 3B and 3C, respectively. It was observed that as the noise level increased, the higher CF responses (above 12) were distorted more severely by noise while lower CF responses (1–12 CFs) were less affected. The effect of noise was quantified by the correlation coefficients (Pearson product-moments) between the neural responses of the clean and the corresponding noisy responses for each CF, and the result is shown in Fig 4. Two levels of noise were considered such as the 0 and 15 dB SNR. The mean and standard deviation of correlation coefficients for five speech samples taken from the YOHO database are shown as a function CF. For clarity, 50% standard deviation values are shown. It is obvious that the lower CFs showed the higher correlation measures for both levels of noise and the responses of higher CFs were more distorted by noise. In other words, the neural responses of the first 12 CFs (up to ~1 kHz) were more robust against noise compared to the responses of other higher-CF fibers. This is also consistent with the observation that even with moderate amounts of noise, the low-energy regions of the speech signal (i.e., high frequency information such as consonants) are substantially modified and cause acoustic mismatch with the clean training data, whereas the high-energy regions (such as the low frequency voiced part of the speech signal) are relatively less affected by noise. Thus, a robust feature extraction scheme must rely on the high energy regions in the spectro-temporal plane. In this study, considering the effects of noise on the neural responses, two different simulation conditions were suggested: (i) using responses of only lower 12 CFs (~<1 kHz) and (ii) using responses of all 25 CFs. It is to be noted that the correlation measure of neural responses for 23–25 CFs to street noise (panel C) was higher compared to those of other noise types. This was due to the fact that the street noise used in this study was band-limited to ~3.7 kHz, and thus the neural responses to clean and noisy signals were highly correlated for CFs beyond that range.

In other words, responses of the first 12 CFs (250 Hz–<1 kHz) should be used for both training and testing under noisy conditions, whereas the responses of all 25 CFs (250 Hz– 4 kHz) should be employed for SI in quiet. So, the dimension of the features used for training and testing in the proposed method would be n by 12 or 25, where n is the number of frames. On the other hand, the number of features in the alternative existing methods was substantially higher, which is described in the next section.

### Existing Features

In this study, the performance of the proposed method was compared to the identification results of three traditional baseline feature-based methods (MFCC, FDLP and GFCC). This section presents a short description of each of the baseline method. To obtain a fair comparison of the performance of different front-end features, only the front-end feature extraction was varied and the configuration of the back-end of the system (i.e., the classifier GMM-UBM) remained the same in all experiments throughout this paper. Also, the performances of all methods were evaluated for two cases: using feature from the narrowband (250 Hz to ~ 1 kHz) and wide-band (250 Hz to ~ 4 kHz) frequencies.

#### MFCC

Mel-frequency cepstral coefficient (MFCC) is a short-time cepstral representation of a speech which is widely used as a feature in the field of speech processing applications such as voice recognition [40], speaker recognition [41], speech emotion detection [42], and gender classification [42]. In this study, the RASTAMAT toolbox [43] was used to extract MFCC features from each speech signal. A Hamming window of length 25 ms (with an overlap of 60% among adjacent frames) was used for dividing the speech signal into frames. The log-energy-based 39 MFCC coefficients were then computed for each frame. This set of coefficients consists of three groups: Ceps (Mel-frequency cepstral coefficients), Del (derivatives of Ceps) and Ddel (derivatives of Del) with13 features for each group.

#### GFCC

The Gammatone filter cepstral coefficient (GFCC) is an auditory-based feature used in speaker recognition. GFCC features can be computed by taking the discrete cosine transform (DCT) of the output of Gammatone filter as proposed by Shao *et*. *al*. [44]. According to the physiological observation, the Gammatone filter-bank resembles more to the cochlear filter-bank [45]. The GFCC-based speaker identification is found to achieve a very robust performance, as presented in [17].

In this study, the procedure provided by Shao *et al*. [44] was used to compute GFCC coefficients for each speech frame. A fourth-order 64-channels Gammatone filter-bank was used to extract GFCC features. Instead of log operation which is commonly used in MFCC calculation, the cubic root was applied to extract GFCC features. According to the observation of [17], most information of 64-dimensional Gammatone features remains in the lower 23-order GFCC coefficients due to energy compaction property of the DCT used in the method. Since the zeroth cepstral coefficient was more susceptible to contamination of noise, 22-dimensional GFCC features were used in the present study to simulate the result. The details of the method can be found in [44].

#### FDLP

Ganapathy and colleagues [16] proposed an auto-regressive-model-based frequency domain linear prediction (FDLP) technique to extract features from the speech signal. FDLP feature was designed based on high-energy peaks in the T-F domain. The derivation of this feature was done in several steps. Initially, the sub-band Hilbert envelopes was derived using frequency domain linear prediction auto-regressive (AR) model. The FDLP envelopes in each sub-band were then integrated in short-term frames (a 25-ms frame size with a shift of 10 ms). These all-pole envelopes from each sub-band were converted to short-term energy estimates. Those energy values across various sub-bands were used as a sampled power spectral estimate for the second AR model. The output prediction coefficients from the second AR model were converted to cepstral coefficients and used for speaker recognition. This feature-based speaker identification method showed improved performance compared to the results from the baseline MFCC features [16]. In this study, thirty-nine (39) FDLP features were computed from each speech frame. The details of the method can be found in [16].

### Speech Dataset

Speech signals from three well-known text-independent databases and one text-dependent database were used in this study to evaluate the performance of the proposed system in SI task. The speech texts were extracted from the TIMIT, TIDIGT, YOHO and UM databases, and the corresponding responses were simulated to extract features from the neural responses. A brief description of each dataset is provided in this section.

#### YOHO

YOHO is a large scale high quality dataset frequently used for the speaker identification and verification systems [46], and this was collected by the ITT (International Telephone & Telegraph) technical institute in 1989 under the contract of US Government [46]. The sampling rate of this dataset was 8 kHz. In this study, 137 speakers (106 males and 31 females) out of 138 speakers in the database were considered (data for one speaker was corrupted and could not be retrieved). Each speaker has four sets of enrollment session with 24 independent utterances (with three two-digits number, e.g., 27-82-39, pronunciation twenty-seven eighty-two thirty-nine) for each enrollment session. For each speaker, eighteen (18) speech samples out of 24 were randomly selected for training, and the rest of the 6 samples were used for testing.

#### TIMIT

TIMIT (Texas Instrument-Massachusetts Institute of Technology) dataset was frequently used for the general linguistic research and also for the text-independent speaker recognition system [47]. TIMIT database is the collection of speech samples from 630 speakers (438 males and 192 females) with eight major American English dialects having 10 different phonetically rich sentences for each speaker. The sampling rate of the TIMIT dataset was 16 kHz. In this study, 100 speakers out of 630 speakers were randomly selected from different regions and dialect combinations. From each speaker, 8 samples were used for training and the remaining 2 samples were used for testing.

#### TIDIGIT

Texas Instruments, Inc. (TI) designed and collected speech data corpus to develop and evaluate the text-independent recognition system for connected digit sequences [48]. The speech signal was sampled at a rate of 20 kHz. There are 55 male speakers and 57 female speakers with 77 digit-utterance samples for each speaker. Speech signals of 40 speakers (20 males and 20 females) out of 112 were randomly chosen in this study. Fifty speech samples were selected for the speaker modeling (training), and the remaining 27 samples for each speaker were used to test the proposed and the existing systems under different noisy conditions.

#### Dataset: UM

Universiti Malaya (UM) dataset is a text-dependent dataset and is an asset of the University of Malaya, Kuala Lumpur, Malaysia. This dataset has been collected for developing and testing the text-dependent speaker recognition systems [27, 28]. In this dataset, speech samples have been collected from 39 Malaysian native speakers (25 males and 14 females) aged between 22 and 24 years. The speech signals were recorded with a sampling rate of 8 kHz in a sound-proof booth which was specially designed for speech recording. Each speaker was asked to say “Universiti Malaya” for 10 times in different recording sessions. In this study, 7 speech samples out of 10 were selected randomly for training and the remaining three speech signals were used to test the performance of the proposed method.

### GMM-UBM as a Classifier

The most successful statistical classifier that can adopt the speaker modeling paradigm is the Gaussian mixture model (GMM) [49]. The component distributions of GMM classifier can represent individual speaker’s phonetic class distribution and provide smooth transition through mixtures by weighting function which makes the system text-independent. The GMM-based modeling in SI system becomes successful when the expectation maximization (EM) [50] algorithm is applied in GMM. GMM parameters are iteratively refined by the EM algorithm that monotonically increases the likelihood of the estimated model for the observed feature vectors.

Sometimes GMM models all speakers except the test speaker, and it is referred to as a universal background model (UBM) and is mostly applied for speaker verification [3]. The GMM speaker modeling is adapted with the UBM-based trained each speakers' data to make the system faster, stable, and having better performances. The advantage of EM is that it can estimate the necessary GMM parameters from a little amount of training data, and the estimated parameters can be adapted to the new data by maximum a-posteriori (MAP) adaptation [51].

In this study, a GMM-UBM classifier with 128 mixture components was used to train the proposed features to generate a model for each speaker. The same classifier with the same number of mixture components was also used to train the MFCC-, GFCC- and FDLP-based SI systems for comparison.

## Results and Evaluations

In this section, the identification results achieved by the proposed SI system are reported. The proposed system was tested both in clean and mismatched conditions. The performance of the proposed method was also compared to the results using the baseline features such as the MFCC, GFCC and FDLP coefficients.

### Experimental Setup

**Fig 5:**
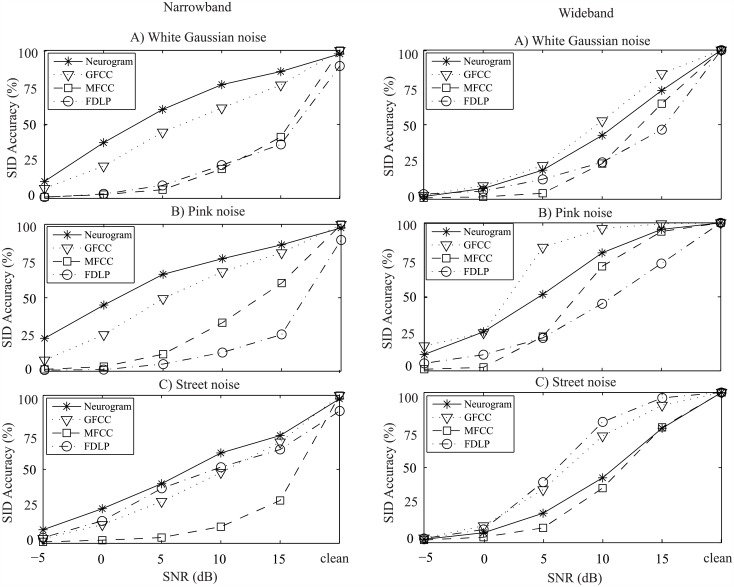
Speaker identification performance of the proposed and existing methods using YOHO database. Two ranges of frequency bands are considered. Left panels: narrowband in which features corresponding to ~<1 kHz are used for SI evaluation; right panels: wideband in which features corresponding to the full range of frequencies (up to ~4 kHz) are considered. Results are shown as a function of SNR with three different types of noise (A: white Gaussian noise, B: pink noise, and C: street noise). Speech samples from 137 speakers were used for evaluation and comparison of the performance of different methods.

**Fig 6:**
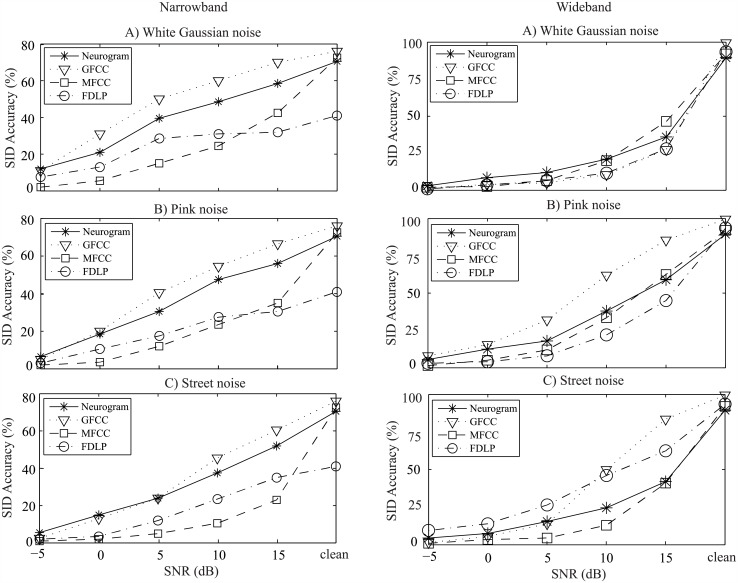
Speaker identification performance of the proposed and existing methods using the TIMIT database. Left panels: Performance is shown for features corresponding to frequencies ~<1 kHz; right panels: features corresponding to the full range of frequencies (up to ~4 kHz) are considered for SI evaluation. Results are shown as a function of SNR with three different types of noise (A: white Gaussian noise, B: pink noise, and C: street noise). Speech samples from 100 speakers were used for evaluation and comparison of the performance of different methods.

**Fig 7:**
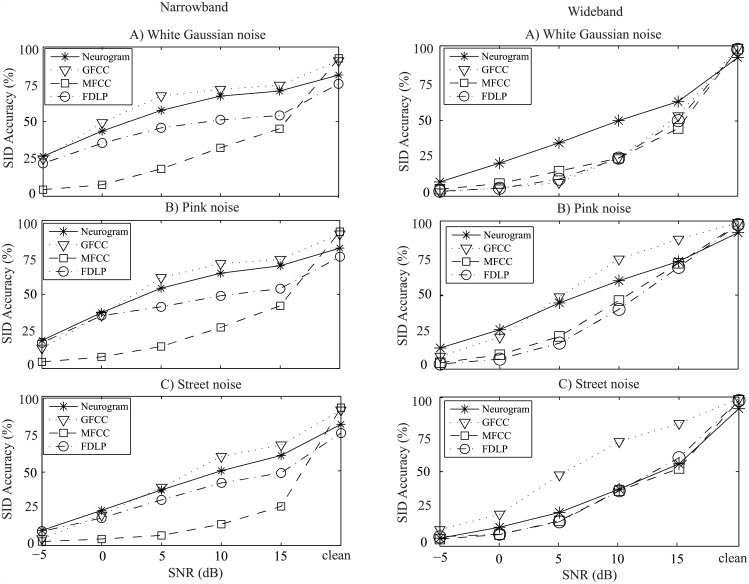
Speaker identification performance of the proposed and existing methods using the TIDIGIT database. Left panels show the SI performances using the features extracted from the lower frequencies (narrowband: <1 kHz), and right panels represent the performances using features from the wideband frequencies. Results are shown as a function of SNR with three different types of noise (A: white Gaussian noise, B: pink noise, and C: street noise). Speech samples from 40 speakers were used for evaluation and comparison of the performance of different methods.

**Fig 8:**
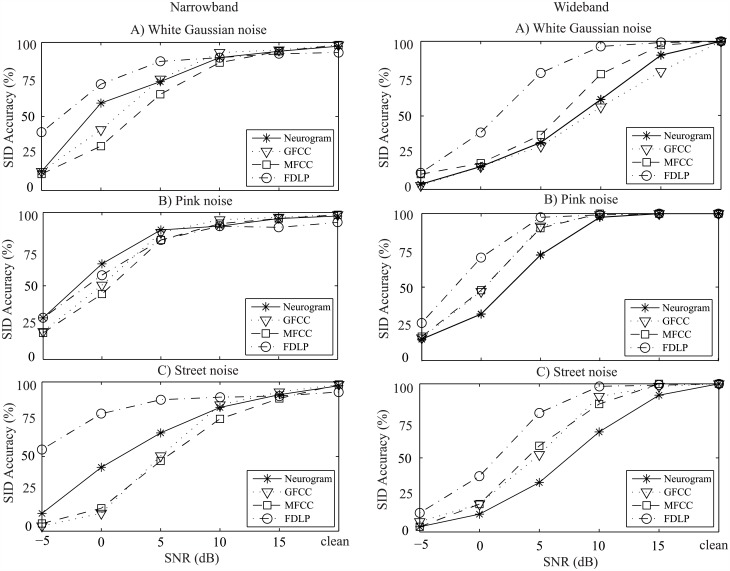
Text-dependent speaker identification performance of the proposed and existing methods using the UM database. Two cases of frequency bands were considered: left panels show the performance of the SI systems using features from the narrowband frequencies (<1 kHz), and the right panels represent performances for the wideband frequencies. Results are shown as a function of SNR with three different types of noise (A: white Gaussian noise, B: pink noise, and C: street noise). Speech samples from 39 speakers were used for evaluation and comparison of the performance of different methods.

The neural responses were simulated to construct neurograms in response to speech signals from the databases. The overall sound pressure level (SPL) of each speech signal was normalized to 70 dB before applying to the AN model. Two cases were considered for the proposed method: using the responses of the first 12 CFs and all (25) CFs for both training and testing phases of the SI system, and the results are shown in Figs 5–8.

In the training stage, generally 70% to 80% of clean speech samples for each speaker were used for GMM-UBM speaker modeling. In the testing stage, the rest of the 20% to 30% of speech samples for each speaker was used directly or corrupted by three types of noise (white Gaussian noise, pink noise, and street noise) with a range of SNRs. The performance of the proposed system was evaluated for three times (by randomly selecting the training and testing samples) for each condition, and it was found that the SI score varied less than 1%.

### Results

Fig 5 shows the text-independent speaker identification results for the YOHO database using the proposed neural-response-based and traditional methods for both narrowband and wideband cases. In general, the performance of the proposed method using the responses from the first 12 CFs (narrowband) was better than that of using responses from 25 CFs (wideband). It is obvious that the performance of the proposed method was comparable to that of other methods in quiet (clean) condition for both narrowband and wideband cases. However, the proposed method showed better identification accuracy compared to other methods under all noisy conditions at low levels of SNR (<0 dB) for narrowband case, as shown in the left panels of Fig 5. On the other hand, GFCC-based method showed better performances under pink and white Gaussian noises for wideband case. The performance of the methods using the baseline features of MFCC and FDLP coefficients declined substantially when noise was added to the clean signal. However, the GFCC feature was found to be superior among the existing features for speaker identification task. Also for wideband case, GFCC resulted better performance compared to the proposed feature when the SNR was higher than 0 dB, as shown in the right panels of Fig 5. For wideband case, FDLP-based method showed the highest performance under street noise condition for SNRs higher than 0 dB, as shown in the lower right panel of Fig 5.

The speaker identification performance of the proposed and several existing methods are shown in Fig 6 for the text-independent TIMIT database. In general, the performance of all methods in quiet was better for wideband case, and the narrowband case was preferable under noisy conditions, especially at lower SNRs. GFCC-based method showed an overall better result for this database. For narrowband case, the performance of the proposed method was comparable to the GFCC-based performance for all types of noise, whereas the MFCC- and FDLP-based methods showed poorer performances at all SNRs studied, as shown in the left panels of Fig 6. However, for wideband case, the proposed method showed poorer results, especially for pink and street noises at moderate levels of SNR (10 and 15 dB). Again, the performance of the existing methods using MFCC and FDLP coefficients for wideband case declined substantially under noisy conditions, although FDLP-based system showed better performance under street noise condition.

The TIDIGIT dataset speech materials were used to test the SI performance of the proposed and the existing methods, and the results are shown for both narrowband (left panels) and wideband (right panels) cases in Fig 7. For the narrowband case, the GFCC-based system showed an overall better performance irrespective of noise types and SNR levels, and the proposed method performance was comparable to those of GFCC-based results. Although both MFCC- and FDLP-based methods performed poorly compared to that of GFCC-based method, FDLP-based method showed a relatively better performance than that of MFCCC-based method. For the wideband case, the proposed method outperformed all baseline performances under white Gaussian noise, whereas GFCC-based performances were better under pink and street noises at moderate levels of SNR.

For the text-dependent UM database, the overall performance of all methods was higher compared to the corresponding results for the text-independent cases, which is shown in Fig 8. Also, FDLP-based method for both narrowband and wideband cases showed a better performance compared to the identification accuracy of other methods for different types of noise. For narrowband case, the performance of the proposed system was better than the results of GFCC- and MFCC-based methods for all types of noises, whereas the proposed method performed poorly for wideband case. It is noteworthy that GFCC-based method showed an overall better performance for text-independent databases, whereas for text-dependent SI system, GFCC-based method performed poorly.

## Discussions

This study investigated the implications of the proposed neural-response-based feature in the speaker identification task both in quiet and under noisy conditions. The neural features were derived from the responses of a physiologically-based model of the auditory periphery. Different databases with different combination of speech materials (sentences, words, and digits) were used to test the proposed SI method. The most important finding of this study was that the proposed neural feature resulted a consistent performance across different types of noise (white Gaussian vs. other noises) irrespective of the speech materials and the duration of the signal for both narrowband and wideband cases, whereas most of the baseline feature-based (such as the MFCC, GFCC and FDLP coefficients) systems produced a relatively consistent performance only for the narrowband case. Based on simulation results, the performance of the proposed system using features from the lower frequencies (narrowband) was relatively better than most of baseline feature-based systems under noisy conditions, especially at negative SNRs.

In this study, the performance of the proposed system was evaluated using two different simulation conditions. The responses of the first 12 CFs (narrowband; 250 Hz–< 1 kHz) or all the 25 CFs (wideband; 250 Hz– 4 kHz) were used. It was found that the responses of higher CF fibers (above ~ 1 kHz) were severely affected by noises as shown in Fig 4 and thus provided a comparatively lower SI performance as shown in the right panels of Figs 5–8. Thus, using only the lower CF responses produced better speaker identification performance under noisy conditions, whereas the performance in quiet was poor. However, the performance of the proposed system was nearly 100% in quiet when all the 25 CF responses were employed for identification. The performance of the proposed method as well as all other existing methods dropped significantly under noisy conditions when higher frequency information was considered for mismatched training and testing conditions (text-independent SI). It can be inferred that the lower frequency responses (<1 kHz) were, in general, less affected by noises employed in this study, and thus the SI system provided a relatively robust performance under noisy conditions. In light of this observation, a pre-processing step (noise/no noise binary classifier rule) can be implemented to improve the performance of the SI system. This can be done, for example, by computing signal energies for the first few frames at the beginning of the input speech signal which normally represents silence periods (speech absence). If the energy estimation lies over a preset value of threshold, then the noise is detected and the speech signal is considered as noisy signal (noisy condition); otherwise, the input speech is treated as a clean signal for the proposed SI system.

Among different types of noises, the responses of lower 12 CFs were relatively more distorted under street noise compared to the degradation observed (in correlation between the noisy and clean responses) for white Gaussian and pink noises, as illustrated in Fig 4. Thus, the performance of the proposed system under street noise was slightly lower across all databases compared to the results for other noises (Figs 5–8). Although the results are relatively consistent across databases, the noticeable differences in SI performance might arise from the variation in speech materials used in the study. For example, the standard deviation of signal length for TIDIGIT dataset was quiet high for different digits alphabets, whereas for YOHO and TIMIT, the variation in length of the speech signal was relatively smaller.

In this study, the performance of the proposed method was evaluated and reported for speech signals corrupted by three different types of additive noise. In order to test the robustness of the proposed method against other types of distortion, the HTIMIT dataset was used to evaluate the speaker identification performance. The HTIMIT corpus is a recording of a subset of the TIMIT corpus through 10 different telephone handsets. Using the same classification technique and experimental setup for TIMIT database, the proposed method produced an accuracy of 94.3% for 100 speakers randomly chosen from the database.

To study the effect of neurogram resolution on speaker identification, three different effective window lengths of 7.6, 12 and 25 ms were considered for smoothing. It was observed that the SI performance was, in general, not substantially different from each other for these window resolutions, although a slightly overall lower performance was seen when a window length of 7.6 ms was used (as shown in Table 1 for YOHO dataset). It is to be noted that the SI result shown in [27] using temporal fine structure (TFS) neurogram was higher compared to the performance using the low resolution envelope neurogram (as employed in this study). However, the results reported in that study was for the text-dependent SI system. The performance of the proposed method with a 12-ms window length was good for text-independent digit-based dataset (as shown in Table 1), but resulted poor performance for the TIMIT dataset. Thus, in this study the effective length of window was set to 25 ms which produced a relatively consistent result across all datasets (text-dependent and text-independent cases).

**Table 1:** The effect of window resolution on the performance of the proposed SI system in quiet and under noisy conditions. The experiment was done with YOHO speech materials taken from the first 32 speakers.

Noise Type	Window Size	SNR					
		-5 dB	0 dB	5 dB	10 dB	15 dB	Clean
White Noise	7.6 ms	27.08	48.77	77.06	89.06	95.83	100
	12 ms	27.08	57.38	79.69	90.63	94.79	100
	25 ms	22.92	55.21	75	85.94	93.75	100
Pink Noise	7.6 ms	25.52	51.04	78.13	87.5	94.27	100
	12 ms	32.81	62.5	85.42	90.1	97.4	100
	25 ms	30.21	58.33	79.17	87.5	94.27	100
Street Noise	7.6 ms	15.52	26.04	48.44	67.71	83.85	100
	12 ms	17.71	32.29	51.56	72.4	84.9	100
	25 ms	15.1	34.38	56.77	69.79	85.94	100

For each frame (~25 ms) of speech signal, the proposed feature dimension was 25 (in quiet) or 12 (under noisy conditions). On the other hand, the size of the baseline features for each frame (effectively 15 ms) was 39, 22, and 39 for MFCC, GFCC, and FDLP, respectively. However, it is to be noted that the AN model used in this study incurs high computational complexity, because it requires to simulate responses for a wide range of AN fibers (12 or 25) from the peripheral auditory system. Thus the proposed system was computationally very expensive. The time required to extract features for an input speech signal of 4.4 s was ~28, 4, 5, and 3 s for the proposed method, MFCC-, GFCC-, and FDLP-based methods, respectively, using a standard computer in the laboratory.

The robustness of the proposed neural-response-based system could lie on the underlying physiological mechanisms observed at the level of the auditory periphery. Since the AN model used in this study is nonlinear (i.e., incorporates most of the nonlinear phenomena), it would be difficult to tease apart the contribution of each individual nonlinear mechanism towards SI performance. However, it would be useful to shed some light on the possible mechanism towards the identification task, especially under noisy conditions. The AN fiber tends to fire at a particular phase of a stimulating low-frequency tone, meaning that it tends to give spikes at an integer time of period of that tone. It has been reported that the magnitude of phase-locking declines with frequency and the limit of phase locking varies somewhat across species, but the upper frequency boundary lies at ~4–5 kHz [52]. Thus, it is not surprising that in Fig 4, the correlation between the noisy and clean responses declines as a function of CF, and the lower CFs (<1 kHz) show higher correlation coefficients due to the phase locking property of AN model.

In general, the intelligibility declines significantly under noisy conditions when speech level is higher than the conversational speech level (~65-70dB) [53, 54]. Therefore, the identification of speech and speaker is expected to be degraded at SPLs higher than normal presentation level. In addition to the broadened bandwidth of the AN fibers at higher levels, the potential mechanism underlying degraded performance at higher levels is also hypothesized to be related to the loss of synchrony capture by the second formant while synchrony to the first formant increases at higher sound levels [26]. The AN model employed in this study successfully captured these phenomena. In order to investigate the effects of supra-threshold nonlinearity on the SI task, the performance of the proposed method was evaluated and compared for YOHO database in quiet at several sound pressure levels such as 40, 70 and 90 dB SPLs (using all the 25 CF responses). It was found that the SI score was the highest (100%) at 70 dB SPL, and the score declined slightly (~99.39%) at 90 dB SPL, whereas at 40 dB SPL, the performance degraded sharply to 54.12%. On the other hand, among the existing features, MFCC and FDLP coefficients are completely independent of the effect of SPL, whereas the GFCC coefficients take into account the effects of loudness [17]. In the present study, the GFCC-based system was also tested for YOHO database in quiet at 40, 60, and 90 dB SPL, and the identification score was found to be 93.5%, 93.5% and 96.5%, respectively.

Frequency selectivity in the inner ear is fundamental to hearing and plays a critical role in the ability to distinguish and segregate different sounds perceptually. This implies that the cochlear-filter bandwidth might have a crucial effect on the speaker identification performance, especially in light of the current debate on human cochlear tuning (humans might have a sharper tuning than the frequency selectivity of most of the laboratory animals often taken as models of human hearing). To address this, the Q_10_ values of the basilar-membrane filter of the AN model were varied to adjust the cochlear-filter bandwidth to half (sharper) and four times (broader) of the normal values. It is to be noted that the tuning parameters (normal values) of the AN model used in this study were implemented based on the physiological data in cats. The performance of the proposed method in quiet was evaluated for YOHO database for three different values of cochlear-filter bandwidths. The obtained SI score was 100%, 99.1%, and 55.3% for normal, half, and four times broader bandwidth, respectively. This clearly suggests an important role of frequency selectivity in the inner ear towards the speaker identification task. However, exploring the detail contribution of each nonlinear phenomenon observed at the peripheral level of the auditory system on SI task is beyond the scope of this study and could be pursued as a future work.

## Conclusions

This study proposed a novel neural-response-based metric for a robust speaker identification system which worked well for both text-dependent and text-independent tasks. The proposed neural feature successfully captured the important distinguishing information about speakers to make the system relatively robust against different types of degradation of the input acoustic signals. The neural feature was extracted from the responses of a physiologically-based model of the auditory periphery. The performance of the proposed method was evaluated in quiet and under noisy conditions, and also compared to the classification accuracy from several existing methods. In general, the existing SI methods using baseline features as well as the proposed method resulted a relatively robust performance under noisy conditions when features were employed from the lower frequencies (<1 kHz). Using simulated responses from the lower CF fibers (<1 kHz), the performance of the proposed method was relatively better than the results of most of the existing methods, especially at negative SNRs. Also, the proposed neural feature provided a relatively consistent performance across different types of noise irrespective of the speech materials used and the duration of the signal for both narrowband and wideband cases. On the other hand, the performance of most of the existing methods was dependent on the type of noise and the database used for wideband case. For the proposed feature, although it was difficult to assess the effect of each individual nonlinear phenomenon observed at the level of the auditory periphery on the identification accuracy, based on simulation results, it can be inferred that they certainly play important roles in the speaker identification tasks.

## References

[1] Campbell JP Jr. Speaker recognition: A tutorial. Proceedings of the IEEE. 1997;85(9):1437–62.

[2] Soong FK, Rosenberg AE, Juang B-H, Rabiner LR. Report: A vector quantization approach to speaker recognition. AT&T technical journal. 1987;66(2):14–26.

[3] Reynolds DA, Quatieri TF, Dunn RB. Speaker verification using adapted Gaussian mixture models. Digital signal processing. 2000;10(1):19–41.

[4] Dehak N, Kenny P, Dehak R, Dumouchel P, Ouellet P. Front-end factor analysis for speaker verification. Audio, Speech, and Language Processing, IEEE Transactions on. 2011;19(4):788–98.

[5] Makhoul J. Linear prediction: A tutorial review. Proceedings of the IEEE. 1975;63(4):561–80.

[6] Chi T-S, Lin T-H, Hsu C-C. Spectro-temporal modulation energy based mask for robust speaker identification. The Journal of the Acoustical Society of America. 2012;131(5):EL368–EL74. 10.1121/1.3697534 22559454

[7] Nakagawa S, Wang L, Ohtsuka S. Speaker identification and verification by combining MFCC and phase information. Audio, Speech, and Language Processing, IEEE Transactions on. 2012;20(4):1085–95.

[8] Zue V, Seneff S, Glass J. Speech database development at MIT: TIMIT and beyond. Speech Communication. 1990;9(4):351–6.

[9] Furui S. Cepstral analysis technique for automatic speaker verification. Acoustics, Speech and Signal Processing, IEEE Transactions on. 1981;29(2):254–72.

[10] Hermansky H, Morgan N, Bayya A, Kohn P. RASTA-PLP speech analysis technique. ICASSP; 1992: IEEE.

[11] Pelecanos J, Sridharan S. Feature warping for robust speaker verification. 2001.

[12] Nikias CL, Mendel JM. Signal processing with higher-order spectra. IEEE Signal processing magazine. 1993;10(3):10–37.

[13] Padilla MT, Quatieri TF, Reynolds DA. Missing feature theory with soft spectral subtraction for speaker verification Interspeech; 2006.

[14] Cooke M, Ellis DP. The auditory organization of speech and other sources in listeners and computational models. Speech communication. 2001;35(3):141–77.

[15] Li Q, Huang Y. An auditory-based feature extraction algorithm for robust speaker identification under mismatched conditions. Audio, Speech, and Language Processing, IEEE Transactions on. 2011;19(6):1791–801.

[16] Ganapathy S, Thomas S, Hermansky H. Feature extraction using 2-D autoregressive models for speaker recognition. Odyssey 2012—The Speaker and Language Recognition Workshop, 2012.

[17] Zhao X, Shao Y, Wang D. CASA-based robust speaker identification. Audio, Speech, and Language Processing, IEEE Transactions on. 2012;20(5):1608–16.

[18] Zilany MS, Bruce IC, Carney LH. Updated parameters and expanded simulation options for a model of the auditory periphery. The Journal of the Acoustical Society of America. 2014;135(1):283–6. 10.1121/1.4837815 24437768 PMC3985897

[19] Zilany MS, Bruce IC. Modeling auditory-nerve responses for high sound pressure levels in the normal and impaired auditory periphery. The Journal of the Acoustical Society of America. 2006;120(3):1446–66. 17004468 10.1121/1.2225512

[20] Zilany MS, Bruce IC, Nelson PC, Carney LH. A phenomenological model of the synapse between the inner hair cell and auditory nerve: long-term adaptation with power-law dynamics. The Journal of the Acoustical Society of America. 2009;126(5):2390–412. 10.1121/1.3238250 19894822 PMC2787068

[21] Miller RL, Schilling JR, Franck KR, Young ED. Effects of acoustic trauma on the representation of the vowel /ε/ in cat auditory nerve fibers. The Journal of the Acoustical Society of America. 1997;101(6):3602–16. 9193048 10.1121/1.418321

[22] Young ED, Sachs MB. Representation of steady‐state vowels in the temporal aspects of the discharge patterns of populations of auditory‐nerve fibers. The Journal of the Acoustical Society of America. 1979;66(5):1381–403. 500976 10.1121/1.383532

[23] Wong JC, Miller RL, Calhoun BM, Sachs MB, Young ED. Effects of high sound levels on responses to the vowel /ε/ in cat auditory nerve. Hearing research. 1998;123(1):61–77. 9745956 10.1016/s0378-5955(98)00098-7

[24] Zilany MS, Bruce IC. Representation of the vowel/ε/in normal and impaired auditory nerve fibers: model predictions of responses in cats. The Journal of the Acoustical Society of America. 2007;122(1):402–17. 17614499 10.1121/1.2735117

[25] Hines A, Harte N. Speech intelligibility prediction using a neurogram similarity index measure. Speech Communication. 2012;54(2):306–20.

[26] Mamun N, Jassim W, Zilany MS. Prediction of Speech Intelligibility Using a Neurogram Orthogonal Polynomial Measure (NOPM). Audio, Speech, and Language Processing, IEEE/ACM Transactions on. 2015;23(4):760–73.

[27] Razali NF, Jassim W, Roohisefat L, Zilany MS. Speaker recognition using neural responses from the model of the auditory system. Intelligent Signal Processing and Communication Systems (ISPACS), 2014 International Symposium on; 2014: IEEE.

[28] Islam M, Zilany M, Wissam A. Neural-Response-Based Text-Dependent Speaker Identification Under Noisy Conditions. International Conference for Innovation in Biomedical Engineering and Life Sciences; 2016: Springer.

[29] Brookes M. Voicebox: Speech processing toolbox for matlab Software, [Mar 2011]. Available: www.ee.ic.ac.uk/hp/staff/dmb/voicebox/voicebox.html. Accessed 1997.

[30] Kiang NY-s. Curious oddments of auditory-nerve studies. Hearing research. 1990;49(1):1–16. 2292492 10.1016/0378-5955(90)90091-3

[31] Oxenham AJ, Shera CA. Estimates of human cochlear tuning at low levels using forward and simultaneous masking. Journal of the Association for Research in Otolaryngology. 2003;4(4):541–54. 14716510 10.1007/s10162-002-3058-y PMC3202745

[32] Shera CA, Guinan JJ Jr, Oxenham AJ. Otoacoustic estimation of cochlear tuning: validation in the chinchilla. Journal of the Association for Research in Otolaryngology. 2010;11(3):343–65. 10.1007/s10162-010-0217-4 20440634 PMC2914235

[33] Pascal J, Bourgeade A, Lagier M, Legros C. Linear and nonlinear model of the human middle ear. The Journal of the Acoustical Society of America. 1998;104:1509–1516. 9745735 10.1121/1.424363

[34] Greenwood DD. A cochlear frequency-position function for several species– 29 years later. The Journal of the Acoustical Society of America. 1990;87:2592–2605. 2373794 10.1121/1.399052

[35] Glasberg BR, and Moore BCJ. Derivation of auditory filter shapes from notched-noise data. Hearing Research. 1990;47:103–138. 2228789 10.1016/0378-5955(90)90170-t

[36] Shera CA, Guinan JJ Jr, and Oxenham AJ. Revised estimates of human cochlear tuning from otoacoustic and behavioral measurements. Proceedings of the National Academy of Science USA. 2002;99:338–3323. 10.1073/pnas.032675099 PMC122516 11867706

[37] Ibrahim RA, Bruce IC. Effects of peripheral tuning on the auditory nerve’s representation of speech envelope and temporal fine structure cues The neurophysiological bases of auditory perception. Springer; 2010 p. 429–38.

[38] Bruce V, Green PR, Georgeson MA. Visual perception: Physiology, psychology, & ecology. Psychology Press; 2003.

[39] Liberman MC. Auditory-nerve response from cats raised in a low-noise chamber. The Journal of the Acoustical Society of America. 1978;63(2):442–55. 670542 10.1121/1.381736

[40] Muda L, Begam M, Elamvazuthi I. Voice recognition algorithms using mel frequency cepstral coefficient (MFCC) and dynamic time warping (DTW) techniques. arXiv preprint arXiv:10034083. 2010.

[41] Martinez J, Perez H, Escamilla E, Suzuki MM. Speaker recognition using Mel frequency Cepstral Coefficients (MFCC) and Vector quantization (VQ) techniques. CONIELECOMP 2012, 22nd International Conference on Electrical Communications and Computers; 2012.

[42] Sato N, Obuchi Y. Emotion recognition using mel-frequency cepstral coefficients. Information and Media Technologies. 2007;2(3):835–48.

[43] Ellis DP. PLP and RASTA (and MFCC, and inversion) in Matlab 2005.

[44] Shao Y, Srinivasan S, Wang D. Incorporating auditory feature uncertainties in robust speaker identification. Acoustics, Speech and Signal Processing, 2007 ICASSP 2007. IEEE International Conference on; 2007: IEEE.

[45] Patterson R, Nimmo-Smith I, Holdsworth J, Rice P. An efficient auditory filterbank based on the gammatone function, MRC Applied Psych. Unit; 1988.

[46] Campbell J, Higgins A. YOHO speaker verification. Linguistic Data Consortium, Philadelphia 1994.

[47] Bimbot F, Magrin-Chagnolleau I, Mathan L. Second-order statistical measures for text-independent speaker identification. Speech communication. 1995;17(1):177–92.

[48] Leonard RG, Doddington G. Tidigits. Linguistic Data Consortium, Philadelphia 1993.

[49] Reynolds D, Rose RC. Robust text-independent speaker identification using Gaussian mixture speaker models. Speech and Audio Processing, IEEE Transactions on. 1995;3(1):72–83.

[50] Bilmes JA. A gentle tutorial of the EM algorithm and its application to parameter estimation for Gaussian mixture and hidden Markov models. International Computer Science Institute. 1998;4(510):126.

[51] Young S, Kershaw D, Odell J, Ollason D, Valtchev V, Woodland P. Hidden Markov Model Toolkit (HTK) Version 3.2. 1 User’s Guide. Cambridge University Engineering Department, Cambridge, MA 2002.

[52] Palmer A, Russell I. Phase-locking in the cochlear nerve of the guinea-pig and its relation to the receptor potential of inner hair-cells. Hearing research. 1986;24(1):1–15. 3759671 10.1016/0378-5955(86)90002-x

[53] Studebaker GA, Sherbecoe RL, McDaniel DM, Gwaltney CA. Monosyllabic word recognition at higher-than-normal speech and noise levels. The Journal of the Acoustical Society of America. 1999;105(4):2431–44. 10212424 10.1121/1.426848

[54] Dubno JR, Horwitz AR, Ahlstrom JB. Word recognition in noise at higher-than-normal levels: Decreases in scores and increases in masking. The Journal of the Acoustical Society of America. 2005;118(2):914–22. 16158647 10.1121/1.1953107

